# Visual Processing by Calretinin Expressing Inhibitory Neurons in Mouse Primary Visual Cortex

**DOI:** 10.1038/s41598-018-30958-w

**Published:** 2018-08-17

**Authors:** Daniela Camillo, Mehran Ahmadlou, M. Hadi Saiepour, Maryam Yasaminshirazi, Christiaan N. Levelt, J. Alexander Heimel

**Affiliations:** 1 Cortical Structure & Function Group, Netherlands Institute for Neuroscience, an institute of the Royal Netherlands Academy of Arts and Sciences, 1105 BA Amsterdam, The Netherlands; 2 Molecular Visual Plasticity Group, Netherlands Institute for Neuroscience, an institute of the Royal Netherlands Academy of Arts and Sciences, 1105 BA Amsterdam, The Netherlands

## Abstract

Inhibition in the cerebral cortex is delivered by a variety of GABAergic interneurons. These cells have been categorized by their morphology, physiology, gene expression and connectivity. Many of these classes appear to be conserved across species, suggesting that the classes play specific functional roles in cortical processing. What these functions are, is still largely unknown. The largest group of interneurons in the upper layers of mouse primary visual cortex (V1) is formed by cells expressing the calcium-binding protein calretinin (CR). This heterogeneous class contains subsets of vasoactive intestinal polypeptide (VIP) interneurons and somatostatin (SOM) interneurons. Here we show, using *in vivo* two-photon calcium imaging in mice, that CR neurons can be sensitive to stimulus orientation, but that they are less selective on average than the overall neuronal population. Responses of CR neurons are suppressed by a surrounding stimulus, but less so than the overall population. In rats and primates, CR interneurons have been suggested to provide disinhibition, but we found that in mice their *in vivo* activation by optogenetics causes a net inhibition of cortical activity. Our results show that the average functional properties of CR interneurons are distinct from the averages of the parvalbumin, SOM and VIP interneuron populations.

## Introduction

Inhibitory neurons in the cerebral cortex have been classified by morphology, immunohistochemistry, electrophysiology, gene expression and connectivity^1–5^. Combinations of features are similar across areas of the neocortex in different mammals, although interspecies and interareal differences exist^6–8^. This conservation suggests the existence of interneuron classes with universal functions across cortical areas and species. Linking class and function has been difficult, but transgenic lines have made this easier. In this way, we have recently learned about the function of groups of inhibitory neurons expressing parvalbumin (PV), somatostatin (SOM) or vasoactive intestinal polypeptide (VIP)^9^. A fourth, widely used, marker used for classifying interneurons is the calcium-binding protein calretinin (CR)^10,11^. In adult rodent cortex, CR expressing cells are almost exclusively GABAergic^12–15^. They comprise 10–30% of all cortical interneurons in mammals and are primarily present in the upper layers of cortex^6,11^. In mouse V1, CR interneurons are the most abundant group in layer 2/3 (42% of GABAergic neurons^14^). Like PV, SOM and VIP classes, they are a heterogenous group^4,5^. In mouse V1, 55% of CR neurons is also SOM-positive and 35% is VIP-positive, while 35% of VIP cells express CR, and 59% of SOM cells express CR^14^.

Despite the common use of calretinin as a marker, it is unknown if the expression of calretinin correlates with a cellular function or response property. The mouse primary visual cortex is an ideal system to investigate this question. Response properties of interneurons in general, and PV, SOM and VIP cells in particular, have already been investigated in this area^16–22^ as has the impact that these groups have on the response of other neurons^23–30^. Moreover, there has been extensive modeling of the role of inhibition in shaping stimulus selectivity in V1^31–33^. Interneurons as a group are less orientation-selective than pyramidal neurons, but subsets of PV^20,34^ and SOM neurons^19^ are selective for orientation. PV neuron responses are on average more suppressed by surrounding stimuli than SOM cell responses^21,35^. It is unknown whether expression of CR also correlates with visual response properties. In macaque and rat, CR neurons preferentially target GABAergic neurons and have been considered disinhibitory^13,36^. In the mouse, a subdivision of bipolar and multipolar CR neurons has been made. Both have a higher probability to inhibit inhibitory neurons than pyramidals cells, and could thus be disinhibitory, but bipolar CR neurons also inhibit multipolar CR neurons and vice versa^37^. It thus remains to be seen if CR neurons as a whole group are disinhibitory in the mouse.

In order to shed light on the response properties of CR neurons we analyzed their activity during visual stimulation, using *in vivo* two-photon calcium imaging in mice. We found the CR+ population to be less orientation selective and less surround-suppressed than the CR− population. Optogenetically activating CR+ cells reduced the gain in visual cortex.

## Results

### Specificity of the CR-cre mouse line

**Figure 1:**
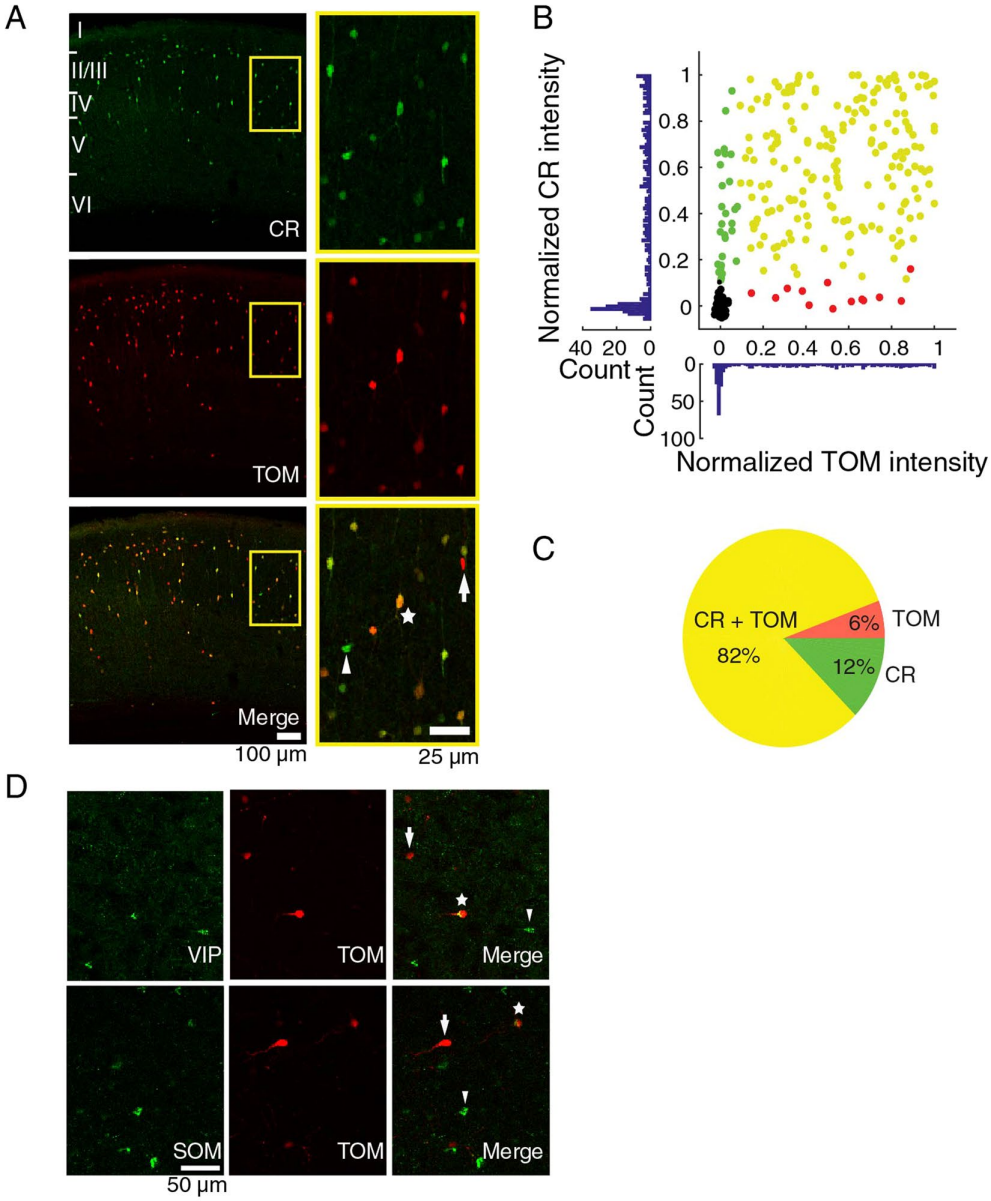
Colocalization of fluorescent label with calretinin. (**A**) Left panels: calretinin (CR) immunoreactive cells (green) and tdTomato (TOM) expressing cells (red) in a coronal slice of visual cortex of a CR-Cre mouse injected with cre-dependent tdTomato virus. Right panels: magnification of left panels. In the lower panel, a TOM−/CR+ cell (arrow head), TOM+/CR+ cell (star), TOM+/CR− cell (arrow). (**B**) Normalized intensities for CR staining and TOM expression. Cells expressing only CR are green, cells expressing only TOM are red, cells expressing both are yellow, and background samples are black. (**C**) Pie chart of the colocalization between CR immunoreactivity and TOM expression. The percentages are of the total number of cells that had TOM and/or CR label. (**D**) Colocalization of TdTomato with VIP and SOM. Higher panel: VIP immunostaining on a coronal slice of a CR-cre mouse injected with a cre-dependent tdTomato virus. From left to right, VIP immunoreactive cells in green, TOM+ cells in red, merge of the two showing a TOM+/VIP− cell (arrow), a TOM+/VIP+ cell (star), a VIP+/TOM− cell (arrow head). Lower panel: SOM immunostaining and colocalization with tdTomato. From left to right, SOM immunoreactive cells (green), TOM expressing cells (red), merge of the previous panels showing a TOM+/SOM- cell (arrow), a TOM+/SOM+ cell (star) and a SOM+/TOM− cell (arrow head).

To visualize CR neurons we used young adult *calb2-ires-cre* mice in which the calretinin promoter drives the expression of cre-recombinase^38^. Different from our previous attempt to specifically target CR interneurons^39^, we injected the mice with a virus driving the expression of the red fluorescent tdTomato protein (TOM) in a cre-dependent way. We first evaluated the specificity of the mouse line by antibody staining against the CR protein (Fig. 1A) and quantifying the colocalization between the TOM expression and the CR immunoreactivity in coronal slices of primary visual cortex. Of all the labeled neurons, 82% were both immunoreactive for CR and expressed TOM (233 cells, 4 slices, Fig. 1B,C). Only 7% of TOM expressing neurons (6% of all labeled cells) did not express CR and 13% of the CR positive neurons did not express TOM (12% of all labeled cells). Considering this high overlap between CR and TOM, we refer to the TOM+ cells as CR+ neurons in the rest of the manuscript. We confirmed that a subset of CR+ neurons also expressed SOM or VIP (Fig. 1D) as expected^14,40^.

### Validation of calcium imaging with cell-attached recordings

**Figure 2:**
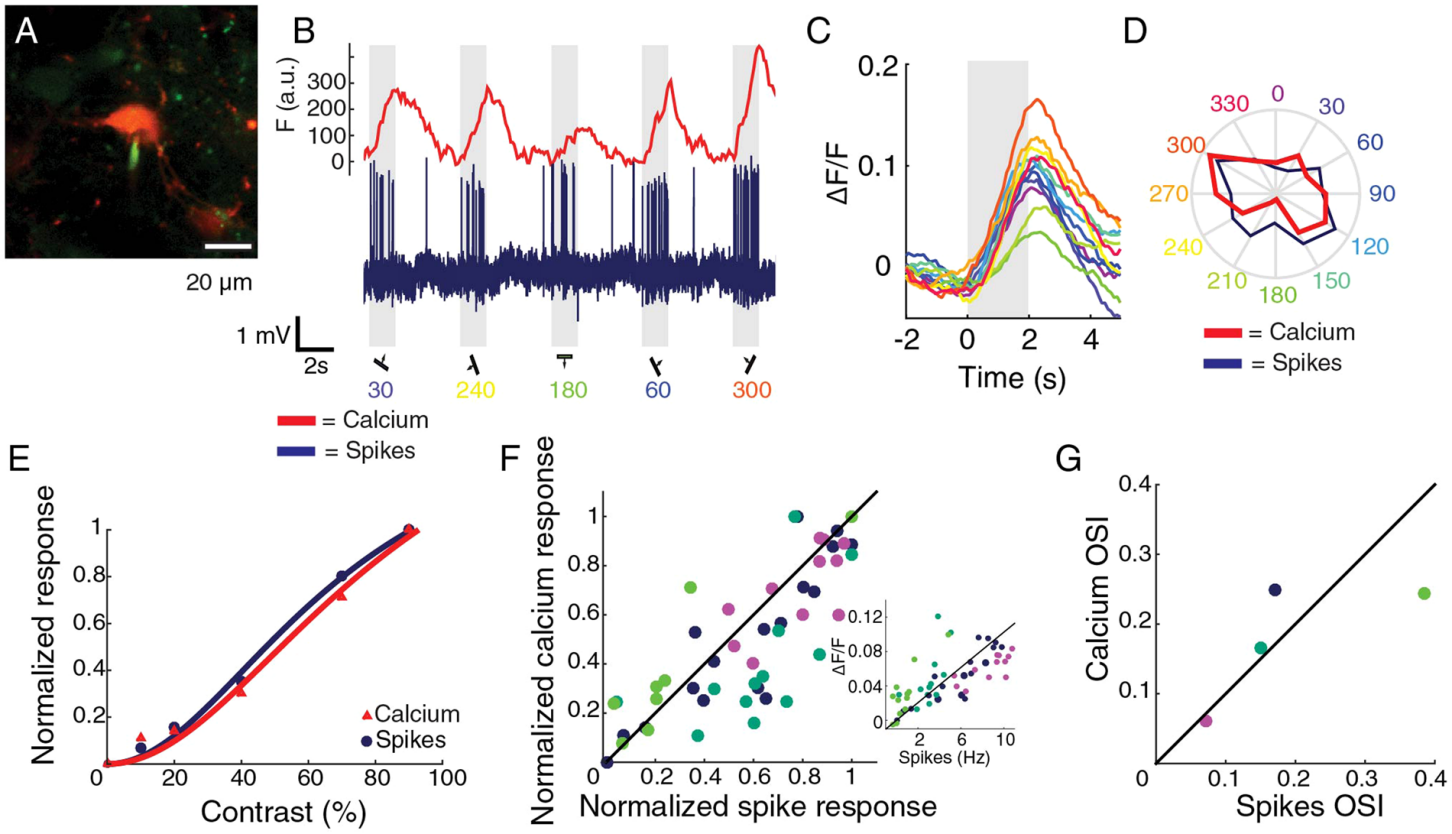
Responses and selectivity measured by calcium fluorescence and cell-attached spiking. (**A**) Example CR+ neuron. In red tdTomato, in green GCaMP6s and dye filled cell-attached pipette. (**B**) Neuronal activity measured as GCaMP6s fluorescence changes (red) and electrophysiological recording (blue) from the cell in A. Grey indicates visual stimulus period. (**C**) Average peristimulus time calcium responses to the different directions for the cell in A. Colors correspond to directions shown in D. (**D**) Polar plot of the responses to the different directions in calcium fluorescence (red) and in spiking (blue) for the cell in A. (**E**) Example normalized responses to different grating contrasts (for cell in **A**). Normalization was done by dividing by the maximum response over all stimuli. (**F**) Correlation between normalized calcium response and normalized spiking response. Each color represents a different cell. Green represents a CR− cell, cyan, blue and magenta CR+ cells. Black line is the identity line. Inset: correlation between the non-normalized calcium response and spike rate. (**G**) Correlation between orientation selectivity indexes computed from calcium and spiking responses. Different colors represent different cells. Green dot represents OSI data for CR− cell, cyan, blue and magenta the OSI for CR+ cells. The identity line is drawn in black.

We injected a virus causing broad expression of the genetically encoded calcium indicator GCaMP6s^41^ to visualize neural activity. The correspondence between changes in fluorescence and electrophysiological responses had been previously shown for pyramidal cells and various classes of interneurons^18,20,42^, but not specifically for CR interneurons. Therefore, we performed two-photon calcium imaging and simultaneously recorded the spiking responses of the imaged neurons in cell-attached configuration (Fig. 2A). We recorded responses to drifting gratings from three TOM+/GCaMP6s+ (CR+) neurons and one TOM−/GCaMP6s+ (CR−) neuron (Fig. 2B). Changes in fluorescence were tightly coupled to electrophysiological spiking activity for different directions (Fig. 2C,D) and different contrasts (Fig. 2E). Across cells, calcium and spiking responses were strongly correlated (54 measurements, 4 cells, 3 mice, Pearson r = 0.82, non-zero slope test p < 10^−7^, Fig. 2F). For the four measured cells the orientation selectivity index (OSI) was qualitatively similar when it was computed from electrophysiological recordings or from imaging (Pearson r = 0.73, p = 0.27, Fig. 2G).

### Reduced orientation selectivity in CR+ neurons

**Figure 3:**
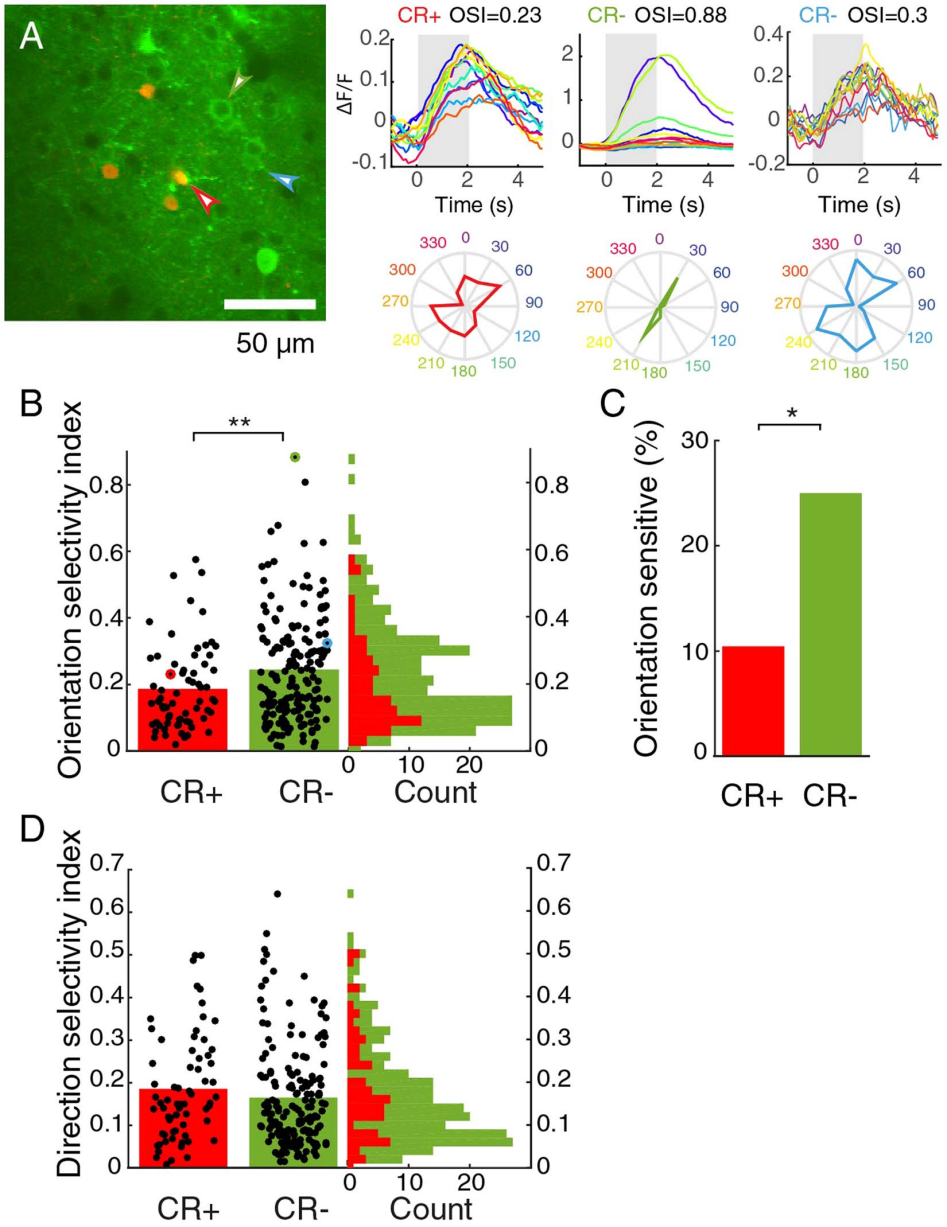
Orientation selectivity is lower in cells expressing CR. (**A**) Two-photon image showing the most orientation-selective CR− cell (green), an averagely tuned CR− neuron (blue) and an untuned CR+ neuron (red). On the right, average responses and polar plots for the same cells. Colors represent stimulus directions. Grey indicates stimulus period. (**B**) Distribution of OSI for CR+ and CR− neurons. Red and green outlined dots are the examples shown in A. Vertical bars show mean. **Indicates p < 0.01. (**C**) Percentage of CR+ and CR− neurons with an OSI higher than 0.33. *Indicates p < 0.05. (**D**) Distribution of DSI is similar for CR+ and CR− neurons. Vertical bars show mean.

Our experiments indicated that changes in fluorescence were a reliable readout of neuronal response. Therefore, we continued to image calcium responses to study the visual processing of CR+ neurons in the mouse primary visual cortex (V1) in more detail. Baseline fluorescence was comparable in the CR+ and CR− group (CR+: mean fluorescence 969 ± 45 arbitrary units, 65 cells; CR−: 1075 ± 58 a.u.; 173 cells, 9 mice, p = 0.64, Mann-Whitney U test). First, we investigated the selectivity and preference of the responses to gratings drifting in different directions (Fig. 3A). The orientation selectivity was lower in the CR+ group than in the CR− group (CR+: mean OSI = 0.18 ± 0.02, 65 cells; CR−: 0.25 ± 0.01, 173 cells, 9 mice, p = 0.0056, Mann-Whitney U test, Fig. 3B). This difference was due to the smaller group of orientation-sensitive cells with an OSI higher than 0.33 (CR+: 11%; CR−: 25%; Chi^2^ test, p = 0.014, Fig. 3B,C). The two groups showed comparable direction selectivity (CR+: mean DSI = 0.18 ± 0.02, n = 65 cells, CR−: 0.17 +/− 0.01, 173 cells, 9 mice; p = 0.32, Mann-Whitney U test, Fig. 3D). Orientation selectivity and direction selectivity were correlated (r = 0.27, p = 0.00034). Both were also slightly correlated with recording depth (both r = 0.2, p = 0.012 uncorrected Chi-square test), but direction and orientation selectivity did not show any significant clustering by depth, which was determined by Hartigan’s dip test on the distribution of the first principal component of depth and each of the selectivity indices (see Methods).

### CR+ neurons are less surround suppressed

**Figure 4:**
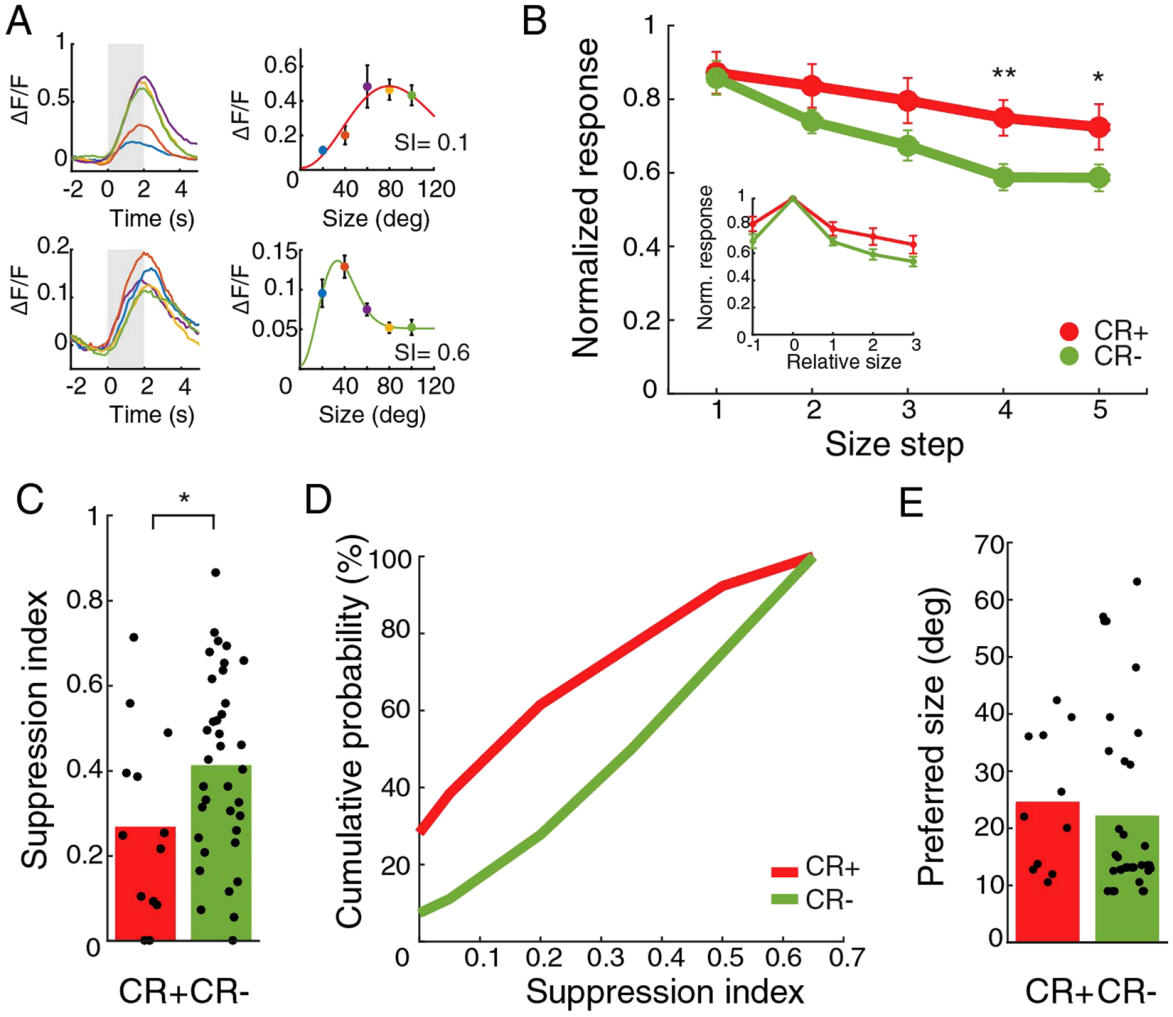
Lower surround suppression in CR+ cells than in CR− population. (**A**) Example average time courses and tuning of responses to stimuli of different sizes for a CR+ (top) and a CR− (bottom) neuron. Grey indicates stimulus period. Colors represent different stimulus sizes. Error bars indicate mean and SEM. (**B**) Normalized response to stimuli of increasing sizes for the CR+ population and the CR− population. Responses were normalized for each cell by dividing the responses by the maximum response over all sizes. *Indicates t-test p < 0.05. **Indicates p < 0.001. In the inset, normalized response to stimuli of size relative to preferred size. Error bars indicate mean and SEM. (**C**) Suppression index for the CR+ population and the CR− population. Bars show mean. *Indicates p < 0.05. (**D**) Cumulative probability profile for CR+ neurons and CR− neurons. (**E**) Preferred diameter for the CR+ group and CR− group. Bars show mean.

After we had determined their preferred direction and their receptive field centers, we continued to study the size tuning of cells (Fig. 4A). On average, cells showed a reduction in response when the grating size becomes large. The CR+ group, however, was less suppressed by large sized stimuli compared to the CR− group (Fig. 4B). To evaluate the response reduction, we computed a suppression index (SI) for all cells. We selected only cells whose preferred orientation differed 30 degrees or less from the presented orientation, and had a receptive field center within roughly 10 degrees (see Methods for details) of the center of the size stimulus. The SI was lower for the CR+ group than for the CR− population (CR+: mean SI = 0.27 ± 0.06, 13 cells; CR−: 0.41 ± 0.04, 36 cells, 4 mice; p = 0.048, t-test, Fig. 4C). The biggest difference was visible at the lower SI range (Fig. 4D). There was no difference in the preferred size (CR+: mean diameter 25 ± 3.6 deg, 11 cells; CR−: 22.2 ± 2.8 deg, 35 cells; p = 0.35, Mann-Whitney U test, Fig. 4E). The combination of the orientation or direction selectivity index and surround suppression index did not reveal any subclusters of CR neurons.

### CR+ neurons have average spatiotemporal frequency and contrast tuning

**Figure 5:**
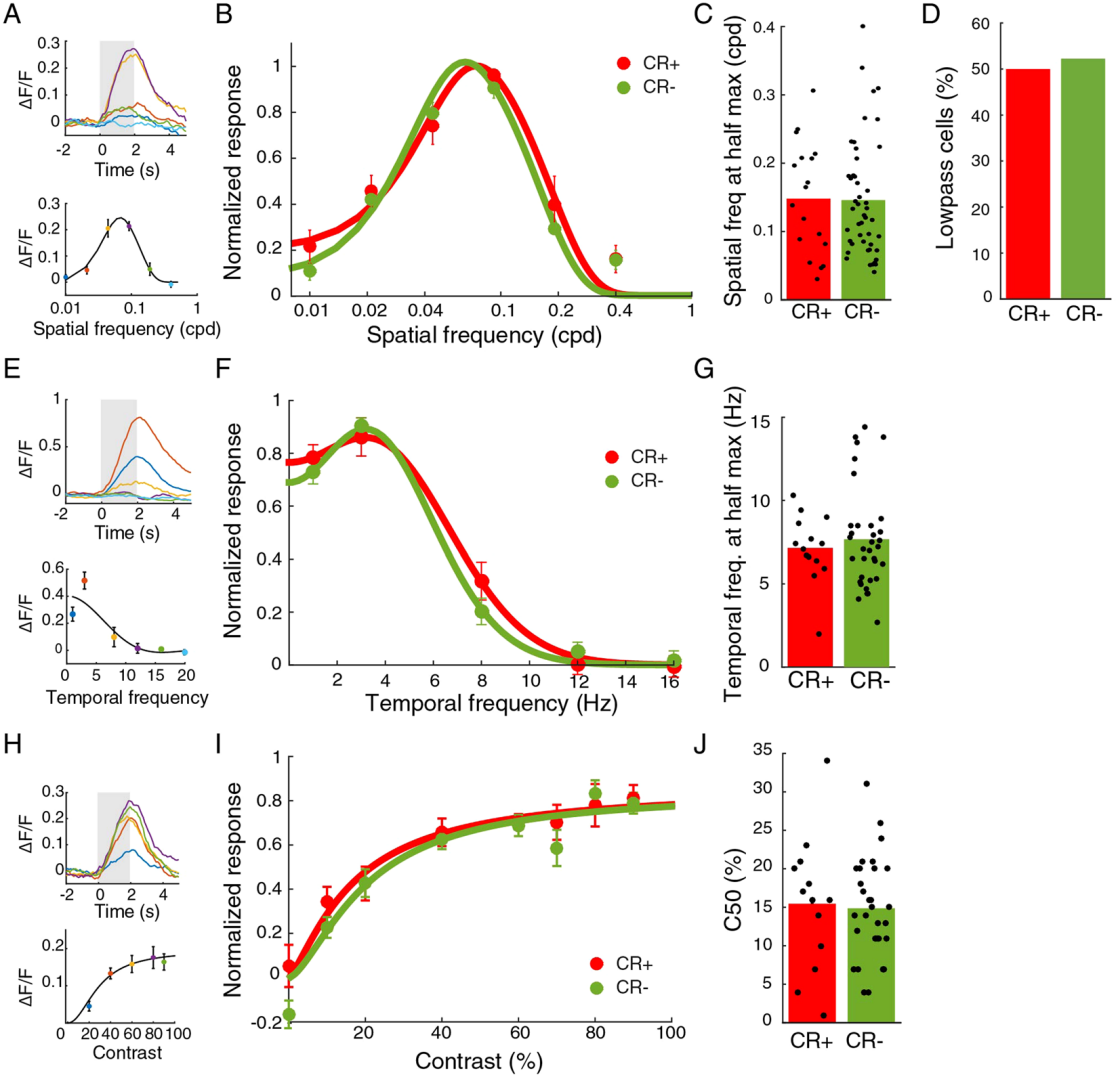
Spatial and temporal frequency and contrast tuning are equal for CR+ and CR− populations. (**A**) Example time courses and mean responses to sinusoidal gratings of different spatial frequency. Colors represent different spatial frequencies. Error bars indicate mean and SEM. Grey indicates stimulus period. (**B**) Normalized responses to various spatial frequency for the CR+ and CR− populations. Responses were normalized for each cell by dividing the responses by the maximum response over all spatial frequencies. (**C**) Means of high spatial frequency at half max. response for CR+ and CR− neurons are equal. Bars show mean. (**D**) Percentages of low-pass cells for the CR+ and CR− groups are equal. (**E**) Example time courses and mean response to stimuli of different temporal frequencies. Colors represent temporal frequencies. (**F**) Temporal frequency tuning for CR+ and CR− populations are similar. Responses were normalized for each cell by dividing the responses by the maximum response over all temporal frequencies. (**G**) Means of high temporal frequency at half max. response for CR+ and CR− neurons are equal. (**H**) Example time course and mean response to gratings of different contrasts. Colors represent contrasts. (**I**) Normalized responses to different contrasts for CR+ and CR− populations are the same. Responses were normalized for each cell by dividing the responses by the maximum response over all contrasts. (**J**) Mean C_50_ values for the CR+ and CR− populations are equal.

We also determined other visual response properties using full screen sinusoidal gratings. The spatial frequency tuning of CR+ and CR− group was very similar (Fig. 5A,B). The high spatial frequency at half maximum response was identical across groups (CR+: mean 0.15 ± 0.02 cpd, 18 cells; CR−: 0.15 ± 0.01 cpd, 47 cells, 8 mice; p = 0.87, Mann-Whitney U test, Fig. 5C). The percentage of low-pass cells was identical (CR+: 50%; CR−: 53%; p = 0.82, Chi^2^ test, Fig. 5D). Also the tuning of CR+ and CR− neurons to gratings drifting at different temporal frequencies was very similar (Fig. 5E,F). The high temporal frequency value at half of the maximum response was not different (CR+: 7.1 ± 0.5 Hz, 14 cells; CR−: 7.7 ± 0.5 Hz, 34 cells, 7 mice, p = 0.88, Mann-Whitney U test, Fig. 5G). In addition, the contrast response curves of the two populations were also similar (Fig. 5H,I). The contrast at half the maximum response (C50) was almost identical in the two cell classes (CR+: 16 ± 2%, 13 cells; CR−: 15 ± 1%, 30 cells, 8 mice; p = 0.8, t-test, Fig. 5J). We looked for possible subclusters in the functional properties of CR cells (described in the Methods), but did not detect any.

### Activating CR+ neurons reduces responses

**Figure 6:**
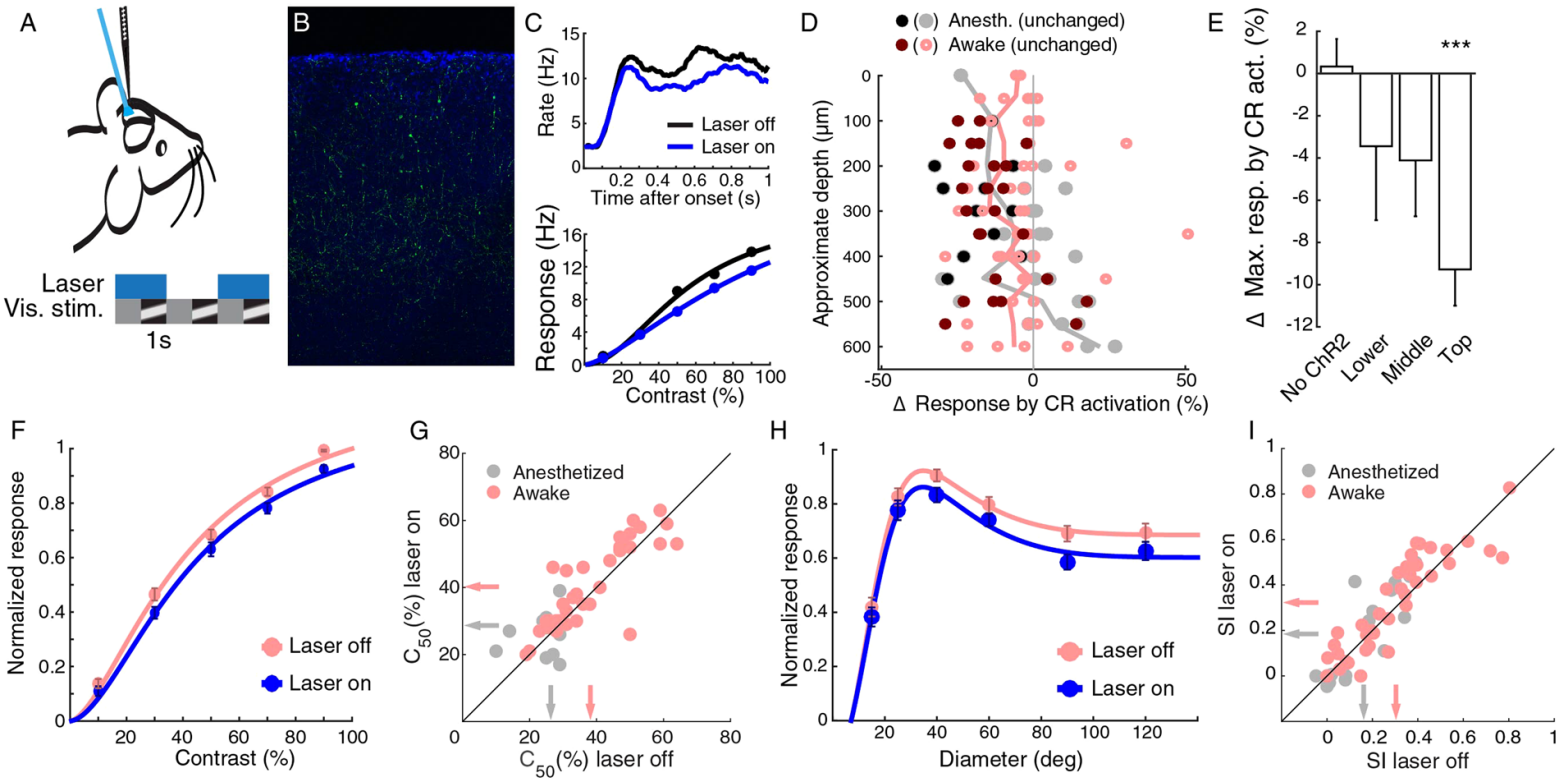
Activation of CR+ cells reduces gain in anesthetized and awake mouse. (**A**) Activation of ChR2 transfected cells by fibre-coupled blue laser. (**B**) Example transfection of ChR2-YFP (green) in DAPI (blue) stained slice of V1. (**C**) Example contrast tuning and spike histogram of cell showing decreased responses when laser is turned on. (**D**) Change in mean responses for contrast tuning curve test for cells when activating CR+ cells for cells in awake (red/pink) and anesthetized (blank/grey) mice plotted versus approximate depth, measured from the top channel in the brain. Dark markers show cells that were significantly modulated (Friedman test), lines show averages per depth. (**E**) Most effect of CR+ activation on mean maximum responses occurs in top layers (awake and anesthetized combined). (**F**) Population contrast tuning curve in top layers with and without CR+ activation in awake mice. Responses were normalized for each cell by dividing the responses by the maximum response over all contrasts for the condition when the laser was off. (**G**) C_50_ in top layers is changed only little by CR+ activation in awake and anesthetized mice. Arrows indicate means. (**H**) Population size tuning curve in top layers with and without CR+ activation in awake mice. (**I**) Surround suppression in top layers is unchanged by CR+ activation. Arrows indicate means.

Next, we wanted to measure the effect that CR neurons exert on the response in the visual cortex. We virally expressed ChR2-YFP in CR+ neurons and used laminar silicon probes to measure multi-unit response to visual stimulation, with and without laser activation of the CR+ population (Fig. 6A–C). Initially, we did this experiment in anesthetized animals and found on average a small, but not significant, reduction in response (−2.8% ± 2.7%, p = 0.39, Wilcoxon signed rank test, 35 units, 5 mice). Under anesthesia, the amount of inhibition compared to excitation is reduced^43^ and it may be more difficult to observe effects. Therefore, we continued the experiments with awake mice. The effect of optogenetically activating the CR+ cells resulted in a stronger reduction in activity (−6.8% ± 1.7%, p = 2 × 10^−7^, Wilcoxon signed rank test, 77 units, 4 mice), but the reduction of activity under anesthesia and in the awake condition did not differ significantly from each other (p = 0.057, Mann-Whitney U-test). Overall, most of the recorded cells showed a reduction in response, although there was a small number of units in the deeper layers that showed an increased mean response to drifting gratings of various contrasts (Fig. 6D). The mean maximum response in the top layer was nearly 10% reduced by laser activation of CR+ neurons (top layer, p < 0.0001, Bonferroni corrected Wilcoxon signed rank test, 49 units; all ChR2 groups, p < 0.0001, Wilcoxon signed rank test, 112 cells, Fig. 6E). There was no significant effect in mice that were not transfected with Channelrhodopsin2 (p = 0.55, Wilcoxon signed rank test, 26 units, 2 mice, Fig. 6E). The difference across layers may be an effect of the reduced amount of laser light reaching the deeper layers. For this reason, we focused for a deeper analysis of the effects on the units in the top layer. Responses were slightly reduced by laser light across the whole contrast response function (Fig. 6F). There was a tiny increase in the mean C_50_, the contrast at half the maximum response of awake mice (awake: 38.2 ± 2.0% laser off, 40.2 ± 2.1% laser on, p = 0.0069, Wilcoxon signed rank test, 37 units, 4 mice; anesthetized: 26.3 ± 2.7% laser off, 28.7 ± 2.9% laser on, p = 0.39, Wilcoxon signed rank test, 12 units, 5 mice, Fig. 6G). We also measured the effect of CR+ activation on size tuning, as there is a group of neurons expressing both somatostatin and CR, and SOM+ neurons are partially responsible for surround suppression^21,27^. As described previously^21,44^, there is more surround suppression in the awake than in the anesthetized animal (awake mean SI = 0.30 ± 0.03, anesthetized 0.16 ± 0.04). CR+ neuron activation only reduced the gain of the responses and did not change the amount of surround suppression (awake laser on mean SI = 0.32 ± 0.03; no effect of laser on SI, p = 0.19, Wilcoxon signed rank test; anesthetized laser on mean SI = 0.18 ± 0.05; no effect of laser on SI, p = 0.49, Wilcoxon signed rank test, Fig. 6H,I).

## Discussion

We studied the functional tuning properties of CR+ neurons in V1 of anesthetized mice. CR+ neurons were less selective to orientation and size than the general population of neurons in V1. CR+ neurons were not less selective for spatial or temporal frequency. The effect of activating CR+ neurons was inhibitory, both under anesthesia and in the awake mouse.

Previously, we and others found that crossing calb2-ires-cre mice with a cre-dependent reporter line caused labeling of many excitatory neurons in the cerebral cortex that only transiently expressed calretinin during perinatal development^5,39,45^. Virus injections of a cre-dependent fluorescent label in adult calb2-ires-cre mice circumvented this problem and worked very well to label cells expressing CR^38^. We found that 93% of the cells that were labeled by tdTomato also expressed CR. This agrees with the specificity of 91% found previously for this calb2-ires-cre mouse line^38^. Possible reasons for cells expressing tdTomato without being marked as CR positive by us could be that these cells expressed CR to a very low degree or that the expression of CR can turn on and off over time, while tdTomato continues being expressed after cre-recombinase had its permanent effect. CR expression in the adult mouse visual cortex is confined to a subset of GABAergic neurons^5,14^. In other species, a minority of CR expression cells in the adult neocortex were not GABAergic (rat^13^, monkey^36^) or formed asymmetric (putative excitatory) connections (rat^15^, human^46^). Calretinin, like other markers for inhibitory neurons, such as parvalbumin, somatostatin and VIP, does not demarcate a completely homogeneous group of interneurons. It is expressed in a subset of somatostatin positive interneurons^4,5,40^, a subset of VIP positive interneurons^4,5^ and a small subset of putative neurogliaform cells positive for neuro-derived neurotrophic factor (Ndnf)^5^. Although the group of calretinin positive neurons thus overlaps with other interneuron classes, it could be that CR+ neurons are still functionally distinct from neurons that do not express calretinin.

Inhibitory neurons in the visual cortex are overall more broadly tuned than excitatory neurons^16,18,47^ and CR+ interneurons are no exception. Mean orientation selectivity is lower than in the general population, as was reported for fast-spiking interneurons, PV, SOM and VIP neurons^17,18,22^. There was a small fraction of orientation sensitive CR+ neurons in our recordings. These could overlap with the orientation selective SOM neurons that were found in the GIN-GFP reporter line^19^. CR+ neurons showed less surround suppression than the general population. This is similar to SOM neurons, and unlike PV neurons^21^. Our data suggest that this broader tuning for orientation and size of calcium responses of CR+ neurons is not due to a different calcium response function. It is known that different neurons can have different relationships between calcium indicator fluorescence and spiking^48^, but we did not see a clear difference in the linearity of calcium responses with respect to spikes for CR+ versus CR− neurons. Furthermore, CR+ neurons were not more broadly tuned in all respects. Direction selectivity, preferred spatial and temporal frequency and the bandwidths for spatial and temporal frequency were not distinguishable from the general population. This also suggests that the lower orientation and size selectivity were not due to a lower firing threshold of the CR+ neurons, but that CR+ neurons really have a different input from CR− neurons. PV interneurons obtain their broad tuning by pooling local inputs from pyramidal cells regardless of their orientation preference^49^. Our data suggest that this may also be the case for CR+ neurons, but the lower size sensitivity also suggests that CR+ neurons integrate inputs over a larger region of visual space, and possibly cortical space, than pyramidal cells and PV interneurons do. The population of CR+ neurons is thus functionally distinct from the PV population by having a lower surround suppression than pyramidal neurons, and from the GIN-GFP SOM population by having a lower orientation selectivity. Although the group of CR+ neurons will contain subclasses of SOM+ and VIP+ cells, we did not find any clustering of functional properties in the CR+ group.

We assessed the response properties under anesthesia. The benefit of this was that there was very little motion artefact and that we did not need to worry about eye position changes between measuring receptive field position and size tuning. One drawback is that anesthesia may mask differences in response properties that are present in the awake animal. For instance, surround suppression is lower under anesthesia^21,44^. And although SOM neurons are active under anesthesia^19,29^, it could be that different neuronal subgroups are affected in different ways by the anesthesia. Sharper differences in the function of these different interneuron classes might also arise during behaviour or learning.

Studies about anatomical connections of CR interneurons showed diversity depending on the model of study. In general, most of the studies showed that CR interneurons mainly target dendrites of other GABAergic cells in the visual cortex of rat^13^. In monkey V1, in layers 1–3 GABAergic neurons are the main target of CR innervation, but in layers 5–6 mostly pyramidal cells are targeted^36^. In humans, GABAergic neurons are not a common target of CR+ axons^46^. In mouse, CR+ neurons (both bipolar and multipolar types) have more than double the probability to connect to a given interneuron than to a given pyramidal cell^37^. For these reasons, CR+ neurons were considered to have a disinhibitory effect in specific cortical layers and areas^10,13,36^. Although a number of cells were disinhibited, we found that the overall effect of activating CR+ neurons was inhibitory and reduced responses. In mouse layer 2/3 CR+ cells can be divided in multipolar CR+ neurons and bipolar CR+ neurons^37^. Multipolar CR+ cells presumably include a subset of SOM positive Martinotti cells and are VIP negative. Bipolar CR+ cells are VIP positive (6 out of 7 tested^37^). Cells in each of these two groups have a much higher chance to be connected to any given cell from the other group than to a cell in their own group. Although the CR connection probability to other inhibitory cells is still greater than to pyramidal cells, the disinhibition of pyramidal cells is offset by the direct extra inhibition pyramidal cells receive from activated CR+ neurons. The combined effect of activating CR+ neurons is thus not disinhibitory in the mouse. The effect of activating the group of CR+/VIP+ bipolar interneurons alone is likely to be disinhibitory, because it was shown that cortical VIP neurons as a group are disinhibitory^50–53^. The optogenetic activation of CR+ neurons that reciprocally inhibit each other is likely to explain the relatively weak effects that we found.

We have established that, on average, CR+ neurons distinguish themselves as a group from excitatory, PV and the overall group of SOM neurons by their visual tuning properties. This is different from the much larger group of neurons that have expressed CR during their lifetime, which could not be distinguished from excitatory neurons by their visual response properties^39^. The CR+ group is, on average, distinct from the overall group of VIP neurons in their effect of activation, because optogenetic activation of CR+ neurons in mice did not increase overall cortical response. The effect might be different in other species like rat (where CR and SOM are not overlapping^54^) and if considering just one of the two cell types (bipolar CR or multipolar CR). It is still unclear why only some neurons express CR. Neither their tuning properties, nor their inhibitory action suggest a particular function for the cells or the protein. Perhaps these functions are occluded by the heterogeneity of calretinin neurons, and further subdividing in VIP+ or SOM+ groups is necessary for this question to be answered. In addition, it may be that the specialty of CR+ neurons only becomes clear during behaviour and learning.

## Methods

### Animals

We used B6(Cg)-Calb2^tm1(cre)Zjh^/J mice of either sex (Jackson laboratory), in which the endogenous Calb2 promoter drives expression of cre-recombinase in calretinin expressing cells^38^. The mice were 1–2 months at the injection and 2–4 months when the recordings were performed. All animals were kept in a 12 hour day/night cycle with access to food and water ad libitum. The experiments were carried out during the day cycle. We maintained body temperature at 36.5–37 degrees C with a heating pad and rectal probe during both surgeries and recordings. All experiments were approved by the institutional animal care and use committee of the Royal Netherlands Academy of Arts and Sciences. All experiments were performed in accordance with relevant guidelines and regulations.

### Virus injections

We anesthetized the mice with isoflurane (1.5–2.5% vol/vol) and administered subcutaneous injections of dexamethasone (4 mg/kg), metacam (1 mg/kg) and amoxicillin (100 mg/kg). We assessed the depth of anesthesia by the pedal reflex. To protect the mice’ eyes, we used cavasan ointment. For calcium imaging, we used a solution containing an equal amount of the adeno-associated viruses containing the vectors Syn.GCaMP6s.WPRE.SV40 and CAG.Flex. tdTomato.WPRE.bGH (University of Pennsylvania Vector Core). For the optogenetic electrophysiology experiments, we used an adeno-associated virus (serotype 9) with a cre-dependent ChR2 expression vector Ef1a.DIO.ChR2.YFP (University of Pennsylvania Vector Core). We injected the viruses in the right V1 in three locations and two depths (200 and 400 µm from dura) centered on stereotactic coordinates 2.9 mm lateral, 0.4 mm anterior to lambda. At each depth of each location, starting at the deepest depth, 10 droplets of 4.6 nl of the virus were injected with 5 seconds intervals using a Nanoject2 volume injection pump (Drummond Scientific Company). Five minutes after the first injection, the pipette was slowly retracted to the upper depth and the same volume was injected again. Five minutes after the injection the pipette was retracted. The scalp was sutured and the mouse was allowed to recover in a warm environment.

### Surgery for calcium imaging

Two weeks after the viral injection, we anesthetized the mice using isoflurane again as described above and we surgically implanted a glass window over a V1 craniotomy^55^. In brief, we applied topical analgesia to the scalp at the start of the surgery with Xylocaine and removed part of the scalp above visual cortex. Next, we attached a coated iron ring with Loctite 454 over V1 to the bone parallel to the plane of the skull and sealed it with black dental cement to reduce the amount of light from the monitor entering the microscope. We drilled a craniotomy of 2 mm diameter and after opening we kept the brain moist with artificial cerebrospinal fluid (ACSF), consisting of a solution of 125 NaCl, 10 Hepes, 5 KCl, 2 MgSO_4_, 2 CaCl_2_ and 10 Glucose, in mM. We filled the space between the dura and the 5 mm glass window with silicon oil (~10 mPa.s viscosity, DC 200, Fluka Analytical, UK) and sealed it with a type 1 glass coverslip (100 µm thickness) and dental cement. In order to later contain the water for the immersion objective of the microscope, we created a well with dental cement. For combined two-photon imaging and cell-attached recordings, we did not use a magnetic ring holder but a metal bar fixed to the mouse stage to allow easier access by the recording pipette.

### Two-photon calcium imaging

We imaged with a converted Olympus BX61WI confocal microscope equipped with a Ti-sapphire laser (Mai-Tai, Spectraphysics, CA, USA), with two non-descanned PMTs with filters optimized for GFP and RFP (Semrock BrightLine FF01-520/70, FF01-625/90 and FF555-Di03 dichroic). We started the imaging sessions 10 days after the surgery and usually did not experience any tissue growth under the glass window. We anesthetized the animals with urethane (1.3 mg/g of mouse body weight); chlorprothixene (8 mg/kg) and injected dexamethasone (4 mg/kg) and atropine sulphate (0.1 mg/kg). The injection of chlorprothixene is routinely used to reduce the amount of urethane needed to anesthetize the mice, and reduces possible urethane-specific effects on brain activity. The mice were head-fixed under the objective using a magnetic holder, connected to the metal ring previously implanted over the skull of the animal (see surgical procedures). A black cloth was used to cover the objective, in order to prevent the light coming from the monitor to reach the PMTs. Before the imaging session we cleaned the window from dust and bedding with 70% ethanol. Two-photon laser scanning microscopy was performed around the area of virus injection, at a wavelength of 910 nm and neurons were imaged using a 20x water-immersion objective (Olympus, 0.95 NA). We scanned at 7 frames per second.

### Two-photon microscopy-guided cell-attached recordings

We followed our procedure as described above and before^29^. In brief, we anesthetized virus-injected animals expressing GCaMP6s in all cells and tdTomato in calretinin expressing neurons with urethane and chlorprothixene and head-fixed the animals using a metal bar. In order for the electrode to reach the brain from the left side, we made a rectangular craniotomy above V1, with the longest side parallel to the recording pipette. We did not use a glass window on top of the brain to leave free access to the pipette. We visualized the neurons expressing tdTomato in the two-photon microscope using a 40x water immersion objective (Olympus, 0.8 NA). We filled a glass pipette (resistance 5–7 MΩ) with a K-gluconate-based internal solution (pH set to 7.3), containing 25 mM Alexa Fluor 488 hydrazide for visualizing the pipette. Under two-photon visual guidance, we brought it close to a target neuron. We applied negative pressure in order to achieve the seal between pipette and cell membrane. We recorded the signals in current clamp mode, lowpass filtered below 5 kHz and digitized at 10 kHz.

### Visual stimulation for calcium imaging

Stimuli were presented on a gamma-corrected Dell UltraSharp U2312HM 23′′ full HD LCD monitor, placed 15 cm in front of the mouse and oriented towards the contralateral eye. Stimuli were drawn by Matlab scripts, available at https://github.com/heimel/InVivoTools, using the Psychophysics Toolbox 3^56^. We first measured orientation tuning using full screen square-wave drifting gratings with different directions going in steps of 30 degrees. Unless otherwise mentioned, the stimulus duration was 2 s, the intertrial stimulus was an isoluminant grey screen of 3 s, contrast was 90%, temporal frequency was 2 Hz and spatial frequency 0.05 cpd. For each test, stimuli were repeated pseudorandomly (i.e. shuffled per block) for 5 minutes. For the two-photon microscopy-guided cell-attached recordings, only 3 repeats were shown per stimulus, except for the orientation tuning measurements of 2 cells, where all stimuli were shown for 6 repeats. Data was analyzed directly after each stimulation block and one responsive CR neuron was chosen for which subsequent stimuli were optimized. The analysis was constrained to neurons with similar response properties to the selected neuron (see next section). Usually, there were a number of such cells per field of view. The center of the receptive field of a neuron was assessed by presenting a drifting grating of the preferred direction in one of 6 × 3 grid locations on the monitor. Next, size tuning stimuli were shown centered at the center-of-mass of the responses of the chosen neuron to all patches, at its optimal direction and at 2 Hz and 0.05 cpd, with fixed diameters corresponding to 20, 40, 60, 80, 100 degrees of visual angle when shown directly in front of the mouse. For Fig. 4E the true sizes were recalculated from the stimulus position. Spatial frequency tuning was assessed using a full screen sinusoidal stimulus of 0.01, 0.021, 0.044, 0.092, 0.191, 0.4 cpd at the optimal orientation for the chosen neuron, drifting at 2 Hz. The physical spacing of the grating was fixed across the screen and the spatial frequency was computed at the center of the screen. Temporal frequency tuning was measured with a full screen sinusoidal grating of 0.05 cpd drifting at 1, 3, 8, 12, 16, 20 Hz.

### Analysis of calcium signals

Circular ROIs were drawn centered in all cells expressing GCaMP6s using Matlab scripts. The changes in fluorescence were divided by the average fluorescence just before stimulus onset to obtain ∆F/F. No neuropil-correction was performed. Response was defined as the average ∆F/F from 0.5 s after stimulus onset to stimulus offset. Cells were said to be responsive if a one-sided t-test of responses versus baseline fluorescence was significant at the 0.1 level. This level best matched human observer judgements of when cells were responsive. Only cells that were responsive and had a maximum response of at least 5% were included in the analysis. Orientation selectivity index was defined as OSI = *√*(*∑R*(*φ*) *sin*(*2φ*)^2^ + *∑R*(*φ*) *cos*(*2φ*)^2^)/*∑R*(*φ*), where *φ* is the angle of the stimulus and *R*(*φ*) the neuron’s response. This is equal to 1 - circular variance. Direction selectivity index (DSI) was defined by DSI = *√*(*∑R*(*φ*) *sin*(*φ*)^2^ + *∑R*(*φ*) *cos*(*φ*)^2^)/*∑R*(*φ*). The suppression index was defined as the (*R*_*preferred*_ − *R*_*largest*_)/*R*_*preferred*_ where *R*_*preferred*_ is the response to the smallest stimulus that reached 95% of the maximum response, and *R*_*largest*_ the response to the largest stimulus^57^. Using the red channel, CR neurons expressing tdTomato were identified. During the experiment one CR cell was chosen to optimize the stimuli. For the analysis of the responses to subsequent stimuli, we selected all cells whose preferred orientation differed 30 degrees or less from the presented orientation, and had a receptive field center within 100 pixels (on a 1920 × 1020 resolution screen, roughly 10 degrees) of the center of the size stimulus. For the spatial frequency tuning we selected cells whose preferred orientation differed 60 degrees or less from the presented orientation. All spatial and temporal frequency curves were fit with a difference-of-gaussian model. From these fits the low and high frequency point at the half maximum responses was interpolated. Cells that did not drop below the half maximum response for low spatial frequencies were considered low pass. Contrast tuning curves were fit with a Naka-Rushton curve and the contrast of half maximum response was taken as C_50_.

To determine if there was clustering of functional properties within the CR neurons, we performed a principal component transform of all pairs and triplets of the following parameters: depth from dura (slightly scattered to remove artificial clustering because cells in a single experiment share their depth), OSI, DSI, SI, spatial frequency at half max., temporal frequency at half max and C_50_. Any clustering would be likely to show up as a dip in the distribution along the first principal component. Therefore, for each pair or triplet, we performed the Hartigan’s dip test on the coefficients of the first principal component. The resulting p-values were Bonferroni corrected for the number of tests.

### Extracellular electrophysiology

Three to five weeks after Channelrhodopsin2 virus injections, we prepared mice for multi-channel extracellular recordings. For the recordings under anesthesia, mice were injected with urethane (1.2 mg/g of mouse body weight) and chlorprothixene (8 mg/kg), and head fixed by ear and bite bars. We injected atropine sulfate (0.1 mg per kg) to reduce mucous secretions. Additional doses of urethane were injected when a toe-pinch response was observed. For awake recordings, mice were first anesthetized with isoflurane (5% induction, 1.2–1.5% maintenance) in oxygen (0.8 L/min flow rate). The eyes were protected from light by black stickers and from drying by Cavasan eye ointment. During the surgery the mice were administered the analgesic Metacam (1 mg/kg) to reduce pain during the recovery. Mice were head fixed and the scalp and soft tissue overlying the skull were incised to expose the skull. A metal ring (5 mm inner diameter) was attached to the skull with glue and dental cement. Small craniotomies for recording were made by dental drill. Next, the head was fixed to a stand through a handle attached to the ring. Animals recovered for two hours before the recordings started. The animals were given water and milk in the first hour after recovery, while they were restrained. Pupil positions were not recorded during the experiments, but tracking the pupil of other mice on the same setup showed no correlation of the movements to this kind of visual stimulation^58^. In both awake and anesthetized animals, laminar silicon electrodes (A1 × 16–5 mm-50–177-A16, 16 channels spaced 50 μm apart, Neuronexus) were used for extracellular recordings from the binocular region of V1 (2900–3000 µm lateral and 300–500 µm anterior to Lambda). The literature and our measurements of receptive field positions showed that this is the binocular region of V1^59^. The signals were digitized at 24 kHz and bandpass filtered between 0.5 and 10 kHz using a Tucker-Davis Technologies RX5 pentusa. Signals were thresholded at 3x standard deviation to isolate spikes, and spikes were sorted by custom-written Matlab (Mathworks) scripts, but single and multi-units were pooled together to increase the number of measurements.

### Visual and optogenetic stimulation for electrophysiology

Stimuli were projected by a gamma-corrected PLUS U2-X1130 DLP projector onto a back projection screen (Macada Innovision, covering a 60 × 42 cm area), positioned 17.5 cm in front of the mouse. Background luminance was 0.1 kcd/m^2^. To determine the receptive field location, we presented a 5 minute movie (5 frames per second) of small white squares (5 deg) in random positions on a black background^60^ (ratio of white to black area: 1/30). The visual stimuli were produced using Psychophysics Toolbox 3^56^. To measure size tuning, we showed circular patches of drifting, square wave, gratings, centered at the most common receptive field location on the probe. The patch diameters were 10, 25, 40, 60, 90 and 120 deg when presented directly in front of the animal. The same physical sizes were also used on the other positions centered on the receptive field of the recording site. The gratings were drifting in 8 different directions (with steps of 45 deg) with 0.05 cpd (in front of the mouse) at 95% contrast. The same physical grating spacing was used for the entire screen. Drift speed was 2 Hz. The stimuli used for measuring contrast tuning consisted of full screen gratings drifting in 8 different directions with 5 different contrasts, 10, 30, 50, 70 and 90 percent. The stimulus and interstimulus time were 1.5 s and 1 s, respectively. The stimuli were presented pseudorandomly, and at least 8 repetitions of each unique stimulus were used to compute responses. An optic fiber connected to a blue laser (473 nm, DPSS Laser BL473T3, Shanghai Laser & Optics Co.) was inserted just above V1 and was used to globally activate the ChR2 while a laminar probe was present in V1. On laser-on trials the light (approx. 2 mW at the tip of the fiber) was turned on at the end of the previous stimulus and remained on during the pre-stimulus period and the entire stimulus. Laser-on and laser-off trials were interleaved in a balanced way.

### Analysis of extracellular electrophysiology

Analysis was done using Matlab scripts (https://github.com/heimel/InVivoTools). For all the analysis of spiking activity for contrast tuning and size tuning stimuli, the responses were averaged over all directions. The rate was the average firing rate during the entire interval that the stimulus lasted (1.5 s). Whenever we use the word response, we refer to the rate minus the spontaneous rate. The spontaneous rate was defined as the mean rate 0.5 s before stimulus onset. Minimum response for a unit to be included was 2 Hz. The surround suppression index and C_50_ were defined the same as for the calcium data. The data was split into three layers. The lowest five sites with visual responses on the probe were labelled deep, the next three sites were labeled middle, and the higher sites were labeled top.

### Immunohistochemistry

After an overdose of pentobarbital (100 mg/kg i.p.), we transcardially perfused the mice with 4% paraformaldehyde (PFA) in PBS, and post-fixated the brains for 2 hours in PFA at 4 °C before moving the brain to a PBS solution. Next, we cut the brains in coronal slices of 50 µm thickness. We incubated the slices for 2 hours in 500 µl blocking solution (0.1% Triton X-100, 5% NGS in PBS) on a rotary shaker at room temperature. We then incubated the slices in 250 µl of primary antibodies, rabbit anti-calretinin (Swant, 1:1000), rat anti-somatostatin (Millipore, 1:200), rabbit polyclonal anti-VIP^61^, 1:1000, in blocking solution per well and left it overnight at 4 °C. The next day we discarded the primary antibody solution and proceeded with 3 washes of 10 minutes at room temperature on the rotary shaker with 500 µl of washing solution (0.1% Tween in PBS). We added 250 µl per well of the secondary antibody solution, goat anti-rabbit Alexa 647 (Life Technologies, 1:700), goat anti-rat Alexa 647 (Life Technologies, 1:700), goat anti-rabbit Alexa 488 (Life Technologies, 1:700) in blocking solution and incubated for 1 hour at room temperature on the rotary shaker. We washed the slices in washing solution 3 times for 10 minutes at room temperature on the rotary shaker. Stained sections were mounted on glass slides with mowiol. For imaging the immunostained sections we used a Leica TCS SP5 Confocal microscope and mainly imaged the superficial layers of primary visual cortex.

### Experimental Design and Statistical Analysis

Values in the text are expressed as the mean ± SEM. For the population statistics of comparing the response properties of CR+ and CR− cells, the distributions were first tested for normality with the Shapiro-Wilk test (at 5% significance level). If both distributions were normal, we used Student t-tests. If one or more of the distributions failed the normality test, we performed the nonparametric Mann-Whitney U-test for two groups. The distribution of activity changes after laser onset over multiple depths failed the normality test, and we used a Kruskal-Wallis test and post-hoc Bonferroni corrected Wilcoxon sign rank test. The distributions in the other comparisons for laser on versus laser off condition also failed the normality tests. In these cases, we have used the nonparametric Wilcoxon sign rank test for paired testing. For determining if individual units were significantly modulated by CR+ activation we used the Friedman test with 5% significance level.

## Data Availability

The datasets generated and analysed for this manuscript are available from the corresponding author.
